# Avicenna aspect of premature ovarian failure

**Published:** 2013-02

**Authors:** Mojgan Tansaz, Roshanak Mokaberinejad, Soodabeh Bioos, Farnaz Sohrabvand, Majid Emtiazy

**Affiliations:** 1 *Faculty of Iranian Traditional Medicine, Traditional Medicine School, Tehran University of Medical Sciences, Tehran, Iran.*; 2 *Department of Iranian Traditional Medicine, faculty of Medical Sciences, Shahed University, Tehran, Iran.*; 3 *Vali-e-Asr Reproductive Health Research Center, Imam Khomeini Hospital Complex, Tehran University of Medical Sciences, Tehran, Iran.*; 4 *Faculty of Iranian Traditional Medicine, Traditional Medicine School, Shahid Sadoughi University of Medical Sciences, Ardakan, Yazd, Iran.*

Dear Editor,

Estrogen deficiency in women is an important risk factor for serious disorders such as severe cardiovascular diseases (1). Several different conditions can lead to estrogen deficiency with premature ovarian failure (POF) being an important one (2, 3). POF includes the cessation of normal ovarian function before age 40, causing menopausal symptoms and general health problems. Although there are several known causes of ovarian failure i.e chromosomal defects, autoimmune disease, exposure to radiation and certain drugs; but most cases of POF are of unknown etiology. Consequently, further work is required to understand the etiology, possible prevention and treatment of POF (4). 

The most influential Iranian physician between 9^th^ and 14^th^ centuries AD was Ibn-Sina or Avicenna (980-1037 A.D). He was a great physician and has written more than 335 books on various subjects. His chief medical book is "Al-Qanon fi Al-Tibb" or "The Canon of Medicine" (5). 

According to "The Canon of Medicine", the basis of health is the right proportion and specific equilibrium of humors (Akhlat) according to their quality and quantity (6). Based on Iranian traditional medicine, there are four humors in the body: "Phlegm, Blood, Yellow bile and Black bile" (7). Each of them is related with a pair of qualities, including cold and wet, hot and wet, hot and dry, and cold and dry, respectively (6).

In Iranian traditional medicine, premature ovarian failure is not defined the same as known today, but in many cases, it has been described as a disorder (8). Due to lack of biochemical analysis of blood parameters, almost all disease states have been defined based on clinical symptoms (9). The most obvious manifestation of POF is amenorrhea (10). The twenty-first chapter of third book of Al-Qanon fi Al-Tibb deals, principally with various kinds of uterine diseases. In this section, amenorrhea is described under a different title: "Ehtebase Tams" which means lack of menstruation. Avicenna has stated that one of the major causes of "Ehtebase tams" is abnormal black bile predominance (8). Based on The Canon of Medicine other symptoms that are seen in the "Ehtebase tams" of abnormal black bile predominance are as follows: vaginal dryness, dry eye, dry skin, anxiety, depression, somatization, sensitivity, hostility, forgetfulness, tiredness, headache, appetite disturbance, sleep disorder and depression and less satisfaction with sexual life (8). New studies have proven all of these symptoms (10). Black bile is divided into two categories: normal and abnormal. In contrast to normal black bile, abnormal black bile has affinity to deposit in any tissue and organ. As a consequence, in cases of abnormal black bile predominance, a high level of its deposition in the ovaries can lead to their dysfunction similar to its effect on other organs and tissues (arteries) (5, 7).

According to this letter, it seems that the treatment of "black bile predominance” can be used as one of the first steps in treating POF patients. This finding can be used as an important theory to design the prevention and treatment plan of POF based on Iranian traditional medicine text books. Most obviously further clinical study is recommended to investigate this issue.
